# Assessment of the Local Exposure Level during Adult Chest X-Rays at the Ngaoundere Regional Hospital, Cameroon

**DOI:** 10.1155/2019/3619498

**Published:** 2019-09-22

**Authors:** Guiswe Gnowe, Henri Paul Ekobena Fouda, Mbo Amvene Jéremie, Takmou Pascal, Bonaventure Babinne Graobe

**Affiliations:** ^1^Higher Institute of Sciences, Health Technics and Management of Garoua, Garoua, Cameroon; ^2^University Technological of Institute, University of Ngaoundere, Ngaoundere, Cameroon; ^3^Faculty of Medicine and Biomedical Sciences of Garoua, Garoua, Cameroon; ^4^General Hospital of Douala, Douala, Cameroon

## Abstract

**Background:**

The purpose of this study was to estimate the doses delivered to adult patients during chest examination for comparison with those elsewhere and to establish a local diagnostic reference level for the chest. The doses delivered in the standard X-ray examinations are not sufficiently optimized and controlled. The working protocols for the same exam given differ for similar morphotypes within the same hospital structure.

**Materials and Methods:**

The entrance skin dose (mGy) of the chest was evaluated on 105 adult patients with a mass of 70 ± 10 kg in accordance with the 75^th^ percentile of the irradiation parameters. The analysis and processing of the data was carried out by Excel 2010. The entrance skin dose of the chest obtained in mGy was 0.18 ± 0.21 for the PA incidence.

**Conclusion:**

The present study allowed us to observe large variations at the entrance skin doses of the chest. These variations have made it possible to understand that the entrance skin doses to the chest are optimized and do not exceed the proportions of those estimated by others and standards internationally. This aspect demonstrates that the diagnostic reference levels as enumerated are dependent on the doses delivered and include not only the notions of quality of the radiographic image and the quality assurance of the radiological equipments but also the level of the manipulators trained.

## 1. Introduction

The medical applications of ionizing radiation have, for many years now, contributed to an improvement of medical practicals and bring a real benefit in terms of health. In radiology, dosimetry and diagnostic quality of images are inseparable. Diagnosis is a function dependent on the quality of the radiological image. Dosimetry could be defined as the measurement of ionizing radiation received or deposited in a medium [1]. Diagnostic medical examinations using ionizing radiation such as radiology, CT, and nuclear medicine lead to variable exposure of patients according to the procedure implemented, the facility of the technology, and the patient's morphotype [2]. In view of this, the reality of these risks, however, comes up against the general problem commonly referred to as the problem of low doses. As observed elsewhere, there are also wide variations in the dose for the same type of examination and the same morphotype of X-ray patients [3]. The health effects of doses delivered in radiodiagnostics are not only low but zero below an imprecise threshold [4] since the linear relationship without threshold not based on 0.6% to 3% of cancers would be attributable to radiodiagnostics.

When one is in a world structured by the important uncertainty of ionizing radiation, uncertainties remain and it is impossible as for the risks created by the ionizing radiations. Some think that the risk exists even at low doses, others think that it does not exist, and others finally think that it can be even more complicated; it is normal that knowledge from all horizons can be taken into account in the definition of protective devices. In the case of ionizing radiation, the difficulty experienced passes from the prevention of proven risks to precautionary approaches aimed at hypothetical risks. This difficulty is evident in the handling of limit values or threshold values considered useful. The radioprotection of patients in imaging appears as an emergency and a particular attention related to the practices because zero risk is hardly possible. Without compromising the effectiveness of the diagnosis or their therapeutic value, the overall goal is to reduce exposure to what is absolutely necessary. That is why any examination must be justified by its diagnostic contribution in relation with the irradiation, its realization must be optimal, that is to say, in conformity with the *ALARA* (*as low as reasonably achievable*) principle, and the doses delivered must be regularly evaluated for comparison with diagnostic reference levels, which should not be exceeded without justification [5]. Regulatory actions, if necessary, must be considered to correct any differences between dose and these irradiation parameters [6]. Moreover, the diagnosis being subordinated by the informative quality of the images may not be applicable but may substitute the DRLs which are indicators of the quality of the practices, allowing each one to situate his practice by the whole of the profession and overexposure of patients.

The use of ionizing radiation for diagnostic or therapeutic purposes is indeed incompatible with a regulatory limitation of doses: the level of irradiation is necessarily subordinate to the medical objective and imposing “a priori” impassable thresholds would be the contradiction detrimental to patients. Radiation protection for people exposed for medical reasons is therefore based exclusively on the principles of justification and optimization [7]. Diagnostic reference levels (DRLs) are defined as “dose levels in medical radiodiagnostic practices, or in the case of radiopharmaceuticals, activity levels, for standard examinations on typical patient groups or on typical ghosts, for broad categories of types of installations” [8]. The Respect for the reference levels is not, by itself, a criterion of good practice. The priority objective, inseparable from dosimetry, is the diagnostic quality of images [9]. This aspect means that the other 25% corresponding to the highest doses were made under nonoptimized conditions. For this purpose, it is necessary to initiate control and correction actions, in case of unjustified overrun of the doses delivered.

Diagnostic reference levels are tools for improving practices and optimizing doses. The Respect for the reference levels is not, by itself, a criterion of good practice. The primary and inseparable goal of dosimetry is to combine the diagnostic quality of images with the reasonably feasible low-dose processes. Recognizing that a diagnostic reference level is a level established for a standard procedure and for typical patient groups and not for individual exposures, compliance with this level does not automatically correspond to the use of good practice, as the quality of the image may be poor and this will not make a good diagnosis. Diagnostic reference levels are of diagnostic interest only if they are assigned to dosimetric quantities accessible by measurement or calculation. Characterizing the level of irradiation of an examination by a different size in conventional radiology and CT may appear as a limitation in a process of justification and optimization of radiological practices [9].

The knowledge of these doses necessarily involves in the determination of doses according to the disymmetric quantities. The determination of these values must be based on the statistical method known as the 75^th^ percentile of the measured dose distribution [10, 11] since 75% of individuals receive doses below these values [7, 11], or the reference levels. Diagnostics are of practical interest only if they are assigned to dosimetric quantities accessible by the measurement. In conventional radiology, the entrance skin dose (ESD in mGy) and the dose area product (DAP in Gy·cm^2^) were retained. The entrance skin dose (ESD) can be determined by two approaches: the indirect method, also called semiempirical method, using the exposure parameters linked to examinations and the direct method using the dosimeter thermoluminescent (TLD) for measurement. These two methods have relatively small differences. The calculation or mathematical method appears reliable and is an effective alternative for measuring the entrance skin dose [12].

The problem of dosimetry usually stems from the incorrect use of radiological equipment and the higher patient exposure required [13]. The optimization and dose determination approach must take into account the specificities associated with the standardization of procedures [14]. The factors influencing the dose delivered to patients in conventional radiology can be classified as follows [10]. Several calculation models are proposed for the evaluation of the dose at the entrance of the skin. But in our approach, we used the estimation of the output [15]. According to the manufacturers, the value of the DAP is also displayed permanently at the control room monitor or actually measured by a device installed at the output of the X-ray tube (ionization chamber) or calculated from the parameters of the exposure and the size of the irradiation field. Because of the two physical laws (inverse of the square of the distance) and geometry (Thales theorem), the value of the DAP is independent of the distance where it is measured [16].

## 2. Materials and Methods

Our study was monocentric and prospective and was carried out at the Radiology and Medical Imaging Department of the Regional Medical Imaging Center of Ngaoundere during the period from April to July 2016. A total of 105 adult patients weighing 70 ± 10 kg who had standard examinations during the study period were involved. The examinations were carried out on a General Electric-branded appliance, model 5192454, whose maximum voltage at the terminals is 150 kV. The studied parameters concerned the patient (age, sex, and anatomical region explored), parameters related to the procedure (focus-film-distance or FFD; focus-skin-distance or FSD; incidence), irradiation (kilovoltage or kV; milliAmperesecond or mAs), and dosimetric constants that were otherwise absent on the manipulative console (entrance skin dose or ESD; the dose area product or DAP).

The first step in calculating the entrance skin dose in standard radiography using the theoretical models is to calculate the power (output) of the ray tube. The power of the tube was estimated in our model study [16] using irradiation parameters directly involved in the achievement of the examinations.

(1) $$\begin{array}{c} \frac{O}{P}\left(\text{mR}\right)=A\times 6.53\times 10^{-4}\left(\frac{\text{mR}}{\text{mAs}}\right){\left({\text{kV}}^{2}\right)}^{-1}\times {\text{kV}}^{2}\times \text{mAs}, \end{array}$$

where (*O*/*P*)mR is the power (output) of the X-ray tube; *A*=0,8, kV is the voltage applied to the tube, for performing the examination; mAs is the charge passing through the tube; *A* was an equal constant of 0.5, 0.8, and 1 for single-phase, three-phase, and high-frequency generator tubes. In our study, the X-ray tube was three-phase. The yields obtained were converted from (mR) to mGy · (mAs)^−1^ by multiplication at a factor of 0.00877/mAs [17].

The entrance skin dose for each patient was calculated using the irradiation parameters of each radiographic examination following this model.

(2) $$\begin{array}{c} \text{ESD}\left(\text{mGy}\right)=A\times 6.53\times 10^{-4}\left(\frac{\text{mR}}{\text{mAs}}\right){\left({\text{kV}}^{2}\right)}^{-1}\times {\text{kV}}^{2}\times \text{mAs}\times {\left(\frac{100}{\text{FSD}}\right)}^{2}\times \text{BSF}\times 0.00877\left(\frac{\text{mGy}}{\text{mR}}\right), \end{array}$$

where  ESD(mGy) is the entrance skin dose, FSD(cm) is the focus-skin-distance, and BSF is the radiation backscatter factor. In the context of this work, it is equal to 1.35 for adults according to the IAEA [1].

The anthropometric data and the technical parameters used (kV, mAs, FFD, and FSD) were collected at the time of examinations. Only images of good qualities having been used for diagnosis were considered. The analysis and treatment of the data according to the 75^th^ percentile of the irradiation parameters as well as the calculation of the entrance skin dose (ESD) of the patients were carried out by Excel 2010.

## 3. Results

The data relating to this examination were collected by means of a questionnaire whose items concerned the equipment, the patient, the radiological technique, the image criteria, and the dose received by the patient. These data were processed and the results obtained are presented in tabular form.  Table 1 presents the distribution of sociodemographic characteristics.  Table 2 shows the performance of the X-ray tube on the chest X-ray.  Table 3 presents the values of the main patient exposure parameters (kV, mAs, FFD, and FSD) used during the course of this review in the study.  Table 4 compares the entrance dose of the chest, the DRLs, and the values found elsewhere.

Table 1 presents the statistical distribution of the sociodemographic characteristics of the patients who participated in this study. Patient anthropometric data (sex, age, and weight) are essential for interpreting the results of radiological examination and dosimetry. Only complete data on 105 adult male and female patients were selected. The average age of men was 40.93, the standard deviation was 7.42 years, and the age range was 29–53 years. In contrast, the mean age of women was 35.58 years, the standard deviation was 10.19 years, and the age range was 23–56 years. Men were more represented than women at 52% or a sex ratio (M/F = 1.1).

Table 2 shows the efficiency of the X-ray tube. These values are essential parameters in the process of optimizing the dose delivered and the quality of the images. They are directly a function of the high voltage (kV) and the load (mAs).

Table 3 summarizes the minimum, maximum, and mean values of the main patient exposure parameters (kV, mAs, FFD, and FSD) used during the course of this exploration in the study.

Table 4 presents a comparative state of entrance skin dose of the thorax, the DRLs, and those obtained elsewhere.

## 4. Discussion

We are very clearly aware of the difficulties of an evaluation without having access to the actual measurement of the dose. It is however necessary to have access to the measured dose to indicate to the user the direction towards which the technical progress is going. Indeed, if these results are closer to the reality of the actual dose, then they can contribute for the more appropriate determination of the “reference” values. From these results, we observe wide variations in the use of pulmonary chest exposure parameters (PA) for patients with similar morphotypes. This diversity of the technical protocols is at the origin of the large variations of the irradiation parameters for the same morphotype. On the contrary, despite the differences observed, our results are less than those estimated in [20], but these are similar to the DRL's for chest radiography. This aspect proves the embryonic state of radioprotection of patients in the radiology departments. These discrepancies with those obtained elsewhere and with international standards can be explained apart by the absence of dosimetric values and working protocols in the examination room.

On the contrary, international standards and DRLs must be used to control and optimize radiological practices. In practice, by displaying and applying these, it is possible to avoid unnecessary irradiation and the periodic evaluation of the delivered doses should become a routine activity. Consequently, the doses delivered for the realization of the thorax are not sufficiently mastered. These differences lead us to think like [5] that unlike developed countries, in sub-Saharan African countries, particularly Cameroon and Mali, legislative and regulatory frameworks are either nonexistent or implemented in a rough way. Moreover, the practice of radioprotection of patients is poorly documented in a context of constant expansion of medical imaging for a decade..

As imaging spreads to the most remote areas of the country, there is an urgent need to optimize work protocols. This optimization of doses delivered to the patients could well be effective by the setting up of a regulatory framework with the obligation of designation and training of humans competent in radioprotection which would allow not only to improve the radioprotection of the patients but also of the personnel, for the establishment of regular dosimetric control and dosimetric standards. The lack of ongoing training of personnel on radiation protection measures could be noted as potential factors of patient exposure. This finding is still very worrying, when the professional profile (nurse and caregivers) of some radiology manipulators does not correspond to their “background” because they are rather converted into radiological manipulators and a large rotation of radiology trainees who find work protocols not available and also who are often forced to produce images capable of being exploited. In view of this, they have either no idea or a rough idea of the good practice in radiodiagnostics or even radiation protection of patients. From these observations, we can affirm with [5] the low level of knowledge in radioprotection in African countries, especially those of sub-Saharan Africa, despite the existence of the laws governing radiation protection and the lack of resources forced to approximate, DRLs.

## 5. Conclusion

At the end of this work, it should be noted that dosimetry is a good match between the image quality and low-dose process. Far from trivializing the exposure of patients to ionizing radiation, it is important to note that the risk of irradiation increases with the nonobservance of the basic principles of radiation protection of patients. The results obtained show that the doses delivered to patients are not optimal. However, an improvement in practices, especially in relation to the technical parameters and protocols associated with strengthening the permanent radiation protection skills of radiological manipulators, could contribute to better radiological protection of patients in this radiology department. Moreover, note that the indirect method used here is a reliable alternative for measuring the entrance skin dose of the patient. This method could also serve as a means of measurement for the control and dosimetric assessment in case of dosimeter deficiency in developing countries whose scarcity of resources constrains the implementation of approximate international conventions and legislation. In radiation protection, however, corrective measures should be evaluated and undertaken, if there are excessive discrepancies between the doses delivered and those observed elsewhere. For this, the radioprotection of patients is an emergency quality in a context where quality is a global aspiration.

## Figures and Tables

**Table 1:** Sociodemographic data.

Radiography	Sex		Age (years)	Weight (kg)
Chest	M	55	29–53 (40.92 ± 7.42)	64–80 (70.84 ± 4.7)
	F	50	23–56 (35.58 ± 10.19)	60–78 (66.66 ± 4.71)
Total		**105**		

M: male. F: female.

**Table 2:** Tube yield in mR and mGy · (mAs)^−1^.

Radiography	Incidence	Output (mR)	Output mGy · (mAs)^−1^
		Min-max	Min-max
Chest	PA	75.22–81.75	0.65–0.71

PA: posteroanterior. Min: minimum. Max: maximum.

**Table 3:** Technical parameters used to conduct this exam.

Radiography	Incidence	kV	mAs	FFD	FSD	ESD		3^rd^ quartile	SD
						Min-max	Max/min		
Chest	PA	120–125 (122.8 ± 1.72)	2.5–4 (3.17 ± 0.46)	140–180 (180 ± 0.09)	160–200 (128 ± 0.20)	0.08–0.09	1.04	0.18	0.21

kV: kilovolt. mAs: milliampere second. FFD: focus-film-distance. FSD: focus-skin-distance. ESD: entrance skin dose. SD: standard deviation.

**Table 4:** Comparison of the entrance skin dose (mGy) with those obtained elsewhere and at DRLs.

Radiography	Incidence	Our study	IRSN (DRL)	Sudan [18]	Iran [19]	Mali [20]
Chest	PA	0.18 ± 0.21	0.3	0.53 ± 0.24	0.70 ± 0.38	3.84

## Data Availability

Our data may be available upon request.
